# Environmental Features Associated With At‐Sea Sightings of Snow Petrel *Pagodroma nivea* in East Antarctica

**DOI:** 10.1002/ece3.71871

**Published:** 2025-08-05

**Authors:** Benjamin Viola, Luke Halpin, Denisse Fierro‐Arcos, Toby Travers, Louise Emmerson, Colin Southwell, Patti Virtue, Natalie Kelly, Stuart Corney

**Affiliations:** ^1^ Institute for Marine and Antarctic Studies University of Tasmania Hobart Tasmania Australia; ^2^ Department of Climate Change, Energy, the Environment, and Water Australian Antarctic Division Kingston Tasmania Australia; ^3^ Gulbali Research Institute Charles Sturt University Albury New South Wales Australia; ^4^ Halpin Wildlife Research Vancouver British Columbia Canada; ^5^ CSIRO Environment Hobart Tasmania Australia

**Keywords:** seabirds, spatial ecology, summer habitat, vessel‐based observations

## Abstract

Over the last 70 years, seabird populations have declined by up to 70%, and represent the most endangered group of birds globally. When compared to other seabird species, there is little known about the Snow Petrel ( *Pagodroma nivea* ) and its marine habitat use—especially in East Antarctica. To better understand what drives Snow Petrel presence within this region, we modeled vessel‐based observations of the Snow Petrel against remotely sensed environmental data using binomial generalized additive models (GAMs). Throughout the 16‐year study period (1991–2006), Snow Petrel presence was associated with areas exhibiting shallower bathymetry, increasing sea‐ice coverage, decreasing sea‐surface height, and increasing wind speed. We then used a subset of the Snow Petrel data to generate a population density map and compare model outputs when data recording methods differ. Specifically, we tested how outputs change when inputs are binomial (presence/absence) versus when inputs include count and effort data. The outputs from both effort‐quantified and presence/absence models identified similar environmental drivers of Snow Petrel presence. However, the effort‐quantified models were more robust, yielding higher deviance explained values and narrower confidence intervals around the environmental variables associated with Snow Petrel presence. Snow Petrel interactions with the tested environmental variables may be driven by associated biological processes—specifically, foraging strategies that target niche areas of high biological productivity in the Southern Ocean. Our study provides an important baseline to compare the likely future distribution of Snow Petrels under different climate change scenarios.

## Graphical abstract

Over the last 70 years, seabird populations have declined by up to 70% across the globe. Compared to other seabirds, little is known about Snow Petrels and their marine habitat use—especially in East Antarctica. This study addresses the former knowledge gap while simultaneously comparing how outputs differ when data collection methods vary.

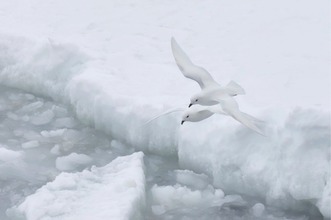

## 1 Introduction

When compared to ecoregions elsewhere in the world, vertebrate diversity in Antarctica is low (Olson et al. 2001; Eastman 2005). However, the continent's unique biotas are subject to international protection under the Antarctic Treaty System (Hughes et al. 2018; Press and Constable 2022). As such, the Antarctic research community and those who oversee governance under the Antarctic Treaty System are responsible for the management of all wildlife—from both a monitoring and a biosecurity perspective (Convey 2011). Area protection constitutes a major mechanism with which these management perspectives can be addressed; and with an emphasis on understanding the habitat needs of species rather than focusing on describing the habitat types of a single location, species‐specific studies are particularly valuable (Phillips et al. 2022).

The Snow Petrel ( *Pagodroma nivea* ) (Forster 1777, Figure 1) is a monotypic seabird confined to polar latitudes in the southern hemisphere (Marchant and Higgins 1990). Much of the ecological research focused on the species has been conducted during the Austral summer, when individuals are present at known breeding locations (Olivier et al. 2004; Viola et al. 2023). Consequently, much of the existing literature focuses on terrestrial aspects of their breeding season (for example: Ryan and Watkins 1989; Chastel et al. 1993; Croxall et al. 1995; Olivier et al. 2004, 2005; Olivier and Wotherspoon 2005, 2006; Thor and Low 2011; Stroeve et al. 2016; Francis et al. 2025) or on palaeoecological deposits (for example: Ainley et al. 2006; Berg, Melles, et al. 2019a; Berg, White, et al. 2019b; McClymont et al. 2022). However, this phase represents only a small part of their overall life history. During the nonbreeding phase, Snow Petrels disperse widely around the Antarctic continent away from their breeding locations—yet their relationship with the marine environment during this time remains understudied (Viola et al. 2023).

**FIGURE 1:**
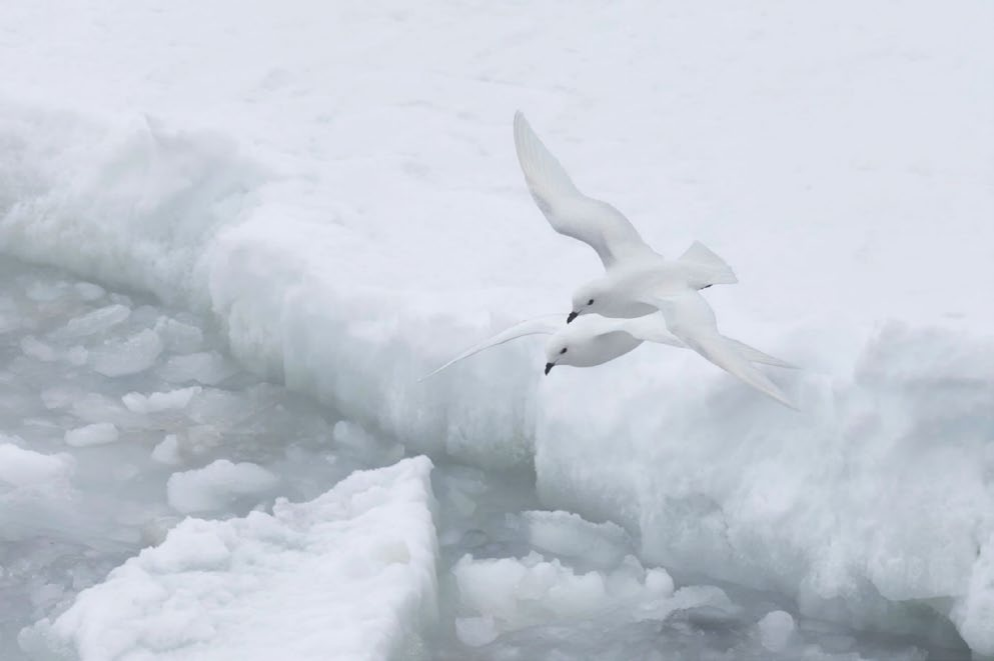
A pair of Snow Petrels ( *Pagodroma nivea* ) near Mawson Station, Antarctica. Taken by Benjamin Viola.

Comparatively fewer studies exist on the marine ecology of Snow Petrels, with the vast majority of research underpinned by visual survey data/at‐sea observations (Ainley et al. 2012). Shipboard surveys have described broadscale seabird community compositions in West (Veit and Hunt 1991; Ainley et al. 1998) and East Antarctica (Veit and Hunt 1991; Woehler et al. 2010). These data have revealed strong associations between many Antarctic seabirds and sea‐ice zones or areas of high biological productivity (Ainley and Jacobs 1981; Ainley et al. 1984, 2017; Fraser and Ainley 1986; Ribic et al. 2011).

Beyond the studies that use at‐sea observation data, few have used tracking devices to model Snow Petrel marine habitat use. At Ile des Pétrels, for example, tracking combined with feather isotope analyses was used to examine the foraging strategies of fulmarine petrels, including Snow Petrels, Southern Fulmars ( *Fulmarus glacialoides* ), and Pintado Petrels ( *Daption capense* ). Snow Petrels occupied the highest trophic level and remained closely associated with sea‐ice habitats during breeding (Delord et al. 2016). Another recent study compared the spatial segregation between sympatrically breeding Antarctic Petrels ( *Thalassoica antarctica* ) and Snow Petrels—finding that Antarctic Petrels generally traveled farther, faster, and longer than Snow Petrels, which were heavily associated with the sea‐ice edge (Phillippot 2024).

Biologging work conducted at Béchervaise Island (Mawson Station) and Filla Island (Davis Station) identified several environmental variables associated with Snow Petrel distribution, including deep bathymetry (> 5000 m), high sea‐ice concentration, low sea‐surface temperature, low sea‐surface height, and proximity to both the Antarctic Circumpolar Current's southern boundary and the sea‐ice edge (Viola et al. 2023). This study revealed diverse inter‐individual movement strategies, with some undertaking (near‐)circumpolar movements during the nonbreeding period, whereas others remained near their breeding colonies. Notably, the drivers behind each of these navigation patterns remain unknown.

Species‐specific insights into Snow Petrel marine habitat use remain limited, particularly in East Antarctica. Beyond understanding the fundamental habitat drivers of Snow Petrel distribution, there is great benefit in researching species through time to enable robust predictions of future distributions as regional climatic conditions change (Olivier and Wotherspoon 2006). Moreover, to understand how contemporary tracking data compare with historical results derived from shipboard surveys, it is important to study Snow Petrels in similar geographic contexts. To date, Snow Petrel outputs derived from shipboard survey data in East Antarctica have been either descriptive or merged with other species' outputs to understand a larger community dynamic (Veit and Hunt 1991; Woehler et al. 2010). Essentially, this means that tracking studies—such as the work at Béchervaise and Filla Islands (Viola et al. 2023)—lack a regional baseline for comparison.

Hereby, in this study, we aimed to build on existing knowledge of Snow Petrel marine habitat use by using shipboard survey data to identify environmental features associated with their at‐sea distribution in East Antarctica. We also examined how variations in shipboard observation methods influence model outputs.

## 2 Methods

### 2.1 Study Area, Data Acquisition and Preparation

All data handling and analysis occurred within the R (v 4.3.3) statistical environment (R Core Team 2023). Results were considered significant where *p* < 0.01 unless stated otherwise.

Seabird observations were recorded during research and resupply voyages by the Australian Antarctic Division (AAD), typically between Hobart, Australia (42.8826° S, 147.3257° E) and three Australian research stations (Casey Station—66.2821° S, 110.5285° E; Davis Station—68.5762° S, 77.9696° E; and Mawson Station—67.6033° S, 62.8742° E) situated along the East Antarctic coastline. Voyages also frequented Australian territories in the Sub‐Antarctic region (Macquarie Island—54.6208° S, 158.8556° E; and Heard Island—53.0818° S, 73.5042° E). Data were acquired and are openly available from the Australian Antarctic Data Centre (https://data.aad.gov.au/).

To ensure methodological consistency and to facilitate spatial overlap between AAD seabird observation data and remotely sensed environmental data, AAD seabird observation data were constrained to the years between 1991 and 2006, inclusive. In total, at‐sea surveys from 136 voyages were used in our final analysis. The combined dataset covers 16 years of oceanic transit and transect data, commencing with the availability of suitable remotely sensed environmental data, and concluding prior to a change in seabird survey method. Shipboard survey efforts commenced when the ship was underway at least once per day during daylight hours, with trained observers recording seabirds within a 300‐m forward quadrant (either port or starboard) of the vessel (as per Woehler et al. 2010).

Due to the vessel routes taken during research and resupply operations, observation data were inherently restricted to waters extending northward from the East Antarctic coastline. To ensure no aberrant extralimital voyages contributed to our dataset, those that ventured beyond the longitudinal range of 0°–180° E were removed. Furthermore, we filtered data from a conservative latitudinal boundary of 30°–70° S. 30° S was chosen as the northern limit in line with unconfirmed vagrant records (*n* = 4 from continental Australia) and the fact that other fulmarine species such as Antarctic Petrel (https://ebird.org/species/antpet1) and Southern Fulmar are occasionally seen at lower latitudes around continental Australia (Vaughan and Viola 2022). The southern limit of 70° S was chosen for this study because no vessel ventured beyond the 70th parallel, and thus no observations existed beyond this point.

Acknowledging that the size and spatial extent of habitat availability can affect coefficient estimates of habitat models (Northrup et al. 2013), we ran the multi‐annual model (see Section 2.2.1) with a dataset subject to tighter boundaries within a latitudinal range of 50°–70° S. This excluded all vagrant observations (*n* = 8). However, model performance was comparable (see Appendices A1, A2) and the interpretation of results did not change. As such, our results represent outputs from the wider latitudinal data.

**FIGURE 2:**
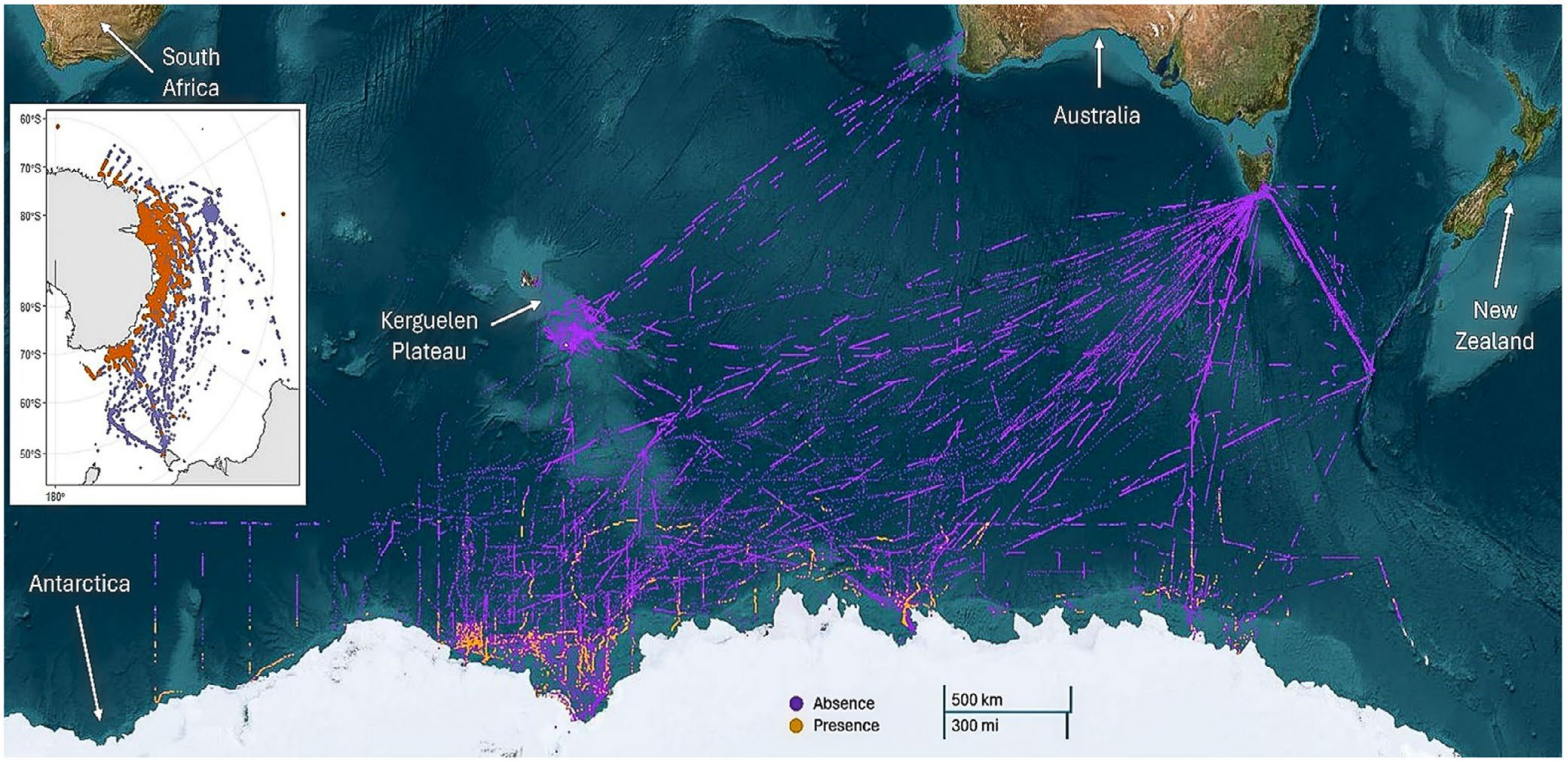
All (*n* = 42,081) presence (orange, *n* = 1009) and true absence (purple, *n* = 41,072) records of Snow Petrel ( *Pagodroma nivea* ) used within the study. Data were collected via shipboard surveys (available via the Australian Antarctic Data Centre). An inlay of sightings without the satellite background image is provided to the left‐hand side of the figure.

Although seabird observation protocols were generally consistent within the specified period (as per Woehler et al. 2010), data recording was not. All records included date, time (UTC), latitude, longitude, and species observed. Data were recorded immediately when a species was observed, and if no species were present, no data were recorded. Seabird data were therefore standardized to the lowest shared granularity: presence or absence observations. Not all voyages recorded survey effort—therefore, absences were inferred from observations where any species was recorded at a given timestamp (±30 s) *without* a concurrent listing of Snow Petrel. After quality control, the final dataset contained 42,081 points, 1009 of which were recorded Snow Petrel sightings and 41,072 were recorded absences (Figure 2).

Environmental variables were chosen in accordance with the available literature on Snow Petrel marine habitat use (Ainley and Jacobs 1981; Ainley et al. 1984, 1998, 2012, 2017; Ribic et al. 2011; Viola et al. 2023), before being scaled and centered. However, additional considerations were made to include variables that capture broader ecosystem functions (as per ‘Justification’ in Table 1). All environmental data were checked for collinearity using Pearson's Correlation Coefficient (PCC) (Pearson and Henrici 1896; Benesty et al. 2009) (see Appendix A3). Variables were inspected for strong pairwise collinearity (i.e., PCC > 0.6) to avoid negative impacts on model performance. Only one such pair was detected: sea‐surface temperature and sea‐ice coverage. We removed sea‐surface temperature from the analysis because sea‐ice: (a) is more variable within the expected marine habitat of Snow Petrels, and (b) has been deemed important to Snow Petrel marine habitat use on the west side of the continent via shipboard survey data (Ainley and Jacobs 1981).

**TABLE 1:** Environmental variables and their raw spatial resolution, justification for inclusion, and model parameters used in the study. All were fit with thin plate regression splines. The number of basis functions was manually calibrated using the *gam.check* function and AIC values (Akaike 2011), to avoid under/overfitting.

Variable	Initial resolution (temporal, spatial)	Justification for model inclusion	Source
Bathymetry (m)	N/A, 30 arc‐second grid	Associated with oceanic physical processes and sea‐ice dynamics (Sun et al. 2022)	GEBCO21 Gridded bathymetry data
Sea‐ice coverage (%)	Daily, 25 km grid	Known to affect abundance and availability of prey in Antarctic ecosystems (Emmerson et al. 2015; Kokubun et al. 2021)	NSIDC Polar stereographic data
Wind speed and direction (m/s)	6‐hourly, 0.25° at 10 m	Ecologically important in ocean systems, particularly for flying seabirds (Mateos and Arroyo 2011; Ainley et al. 2015)	NOAA NCEP‐DOE Reanalysis 2
Eddy kinetic energy (cm^2^/s^2^)	Daily, 0.25°	Important driver of ecosystem diversity, associated with ocean stratification and temperature (Uchida et al. 2019; Rufino et al. 2022)	Derived following Viola et al. (2023)
Sea surface height (m)	Daily, 0.25°	Associated with oceanic surface pressure, and other physical factors such as upwelling (Sokolov and Rintoul 2007)	SSALTO/DUACS experimental products distributed by AVISO + with support from CNS
Sea surface height anomaly (m)	Daily, 0.25°	Associated with the presence of polynyas (Hirabara et al. 2012; Campbell et al. 2019; Narayanan et al. 2024)	Derived following Viola et al. (2023)
Chlorophyll‐*a* (mg/m^3^)	Monthly, 4 km grid	Indicative of primary production (photosynthetic plankton) and a driver for prey fields (though decreasingly so towards higher latitudes) (Hunt et al. 2021; Vereshchaka et al. 2022)	Orbital Sciences Corporation SeaWiFS data

Environmental data were then reprojected to an equal area polar stereographic projection (EPSG 3976) and resampled at a 0.1° resolution using the ‘raster’ package (Hijmans et al. 2015) to match the highest resolution satellite product and to correct for differing geographical distances represented by unit degrees of longitude and latitude.

To test for spatial autocorrelation in detection data, we fit an exploratory model using the ‘sdmTMB’ package (Anderson et al. 2025). Unfortunately, these models do not currently allow for NA values, and whilst interpolation is an option, almost half of our data (~20,000 rows) included missing environmental data for one or multiple variables at any given timestamp due to lack of satellite coverage (e.g., due to cloud cover). We therefore ran the model with complete cases and determined that, despite spatial correlation extending 1369.64 units (on a cutoff value of 200) before decaying, it did not affect model convergence (see Appendix A4). The broad trends depicted by the sdmTMB outputs were later comparable to the multi‐annual model (see Appendix A5), which gave us confidence in our interpretation.

Noting that the sdmTMB output was created using a subset of the data, we visualized the residuals spatially using the complete dataset (Appendix A6) to assess the potential consequences of spatial autocorrelation in the multi‐annual model. Interpolated residuals were calculated using Akima bilinear interpolation (Akima 1978) to provide a continuous surface of model performance across the study area. Although some areas showed localized overprediction of presence (indicated by negative residual values), the majority of interpolated residuals were close to zero, suggesting an overall good model fit. Notably, the spatial pattern of residuals highlights regions where the model may systematically under‐ or over‐predict Snow Petrel presence. Although we did not identify any major concerns arising from this pattern, we encourage readers to interpret our results and discussion within the context of this model fit.

For the multi‐annual model, the spatial components of longitude and latitude were considered as elements of a tensor product smooth within the model, but they were highly correlated with other explanatory variables, and it was deemed that their inclusion would not reveal anything particularly novel about Snow Petrel distribution or their habitat (i.e., reliable information suggests that Snow Petrels are latitudinally constrained, and environmental conditions become more homogenous as they approach the pole (Marchant and Higgins 1990; Menkhorst et al. 2017)). Whilst it is important to include spatial terms to account for any effects of the geographical distribution of the data, these are effectively captured in a biologically interpretable way by the inclusion of sea‐ice concentration as this has a latitudinal element that is well demonstrated and supported elsewhere (e.g., Simmonds 2017). We therefore decided to omit latitude and longitude from the analyses to better understand the habitat features Snow Petrels specifically associate with.

### 2.2 Statistical Analyses

#### 2.2.1 Question 1: Which Environmental Features Are Associated With Snow Petrel At‐Sea Distribution Data in East Antarctica?

**FIGURE 3:**
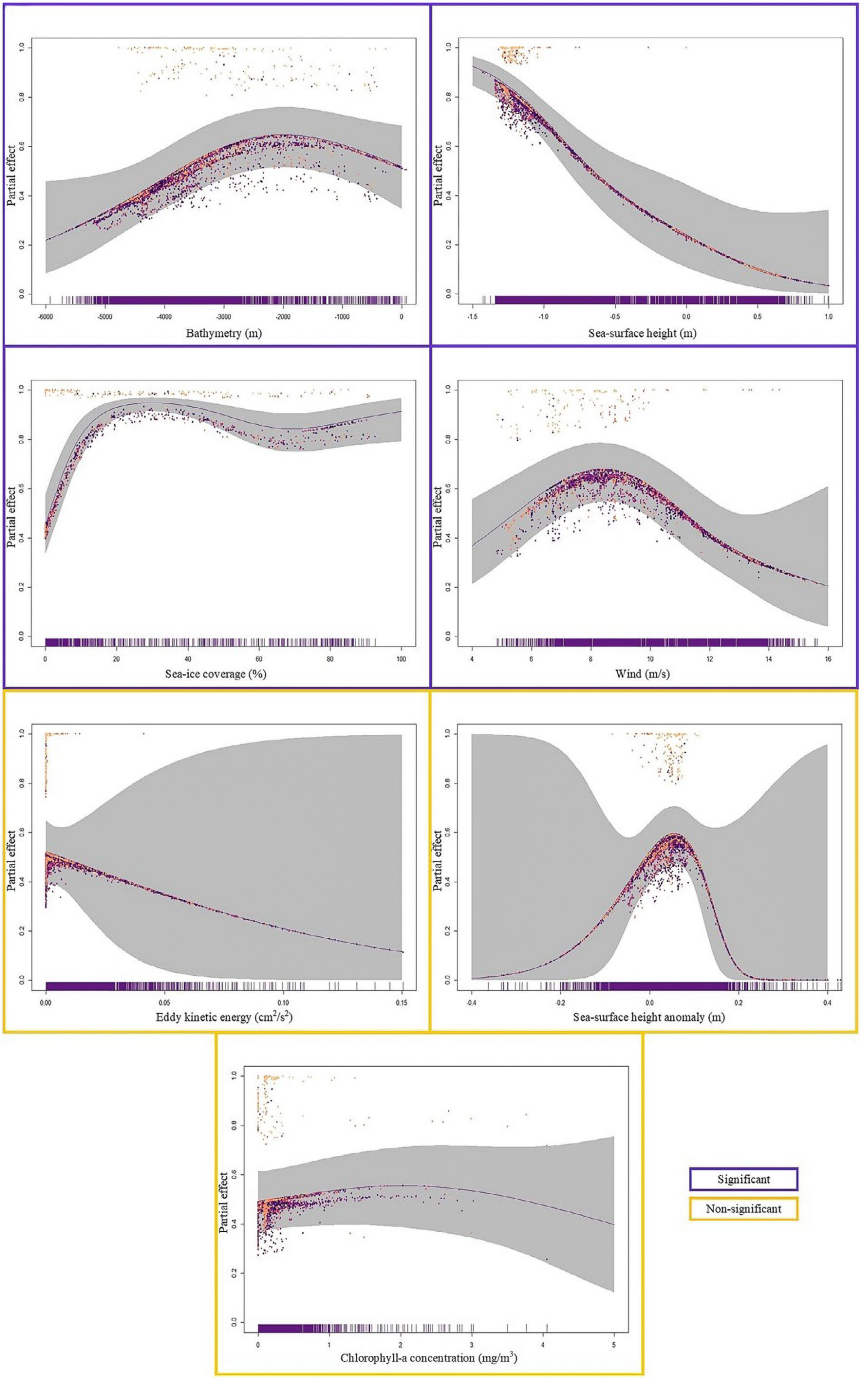
Relative likelihood of Snow Petrel ( *Pagodroma nivea* ) occurrence (partial effect) with regards to environmental variables as determined by a generalised additive model (projected on the probability scale). A 95% confidence interval has been included as grey shading behind the line of best fit. To visually distinguish where different residual values occur, the viridis colour blindness scale has been applied such that lower latitudes are towards the yellow end scale, and higher latitudes are towards the purple end (the model inputs were binary—and when plotting the partial residuals of binary [and Bernoulli] models, two distinct bands will inherently appear [Wood 2017]). Variables with a significant effect on presence are bordered in purple. Those that showed no meaningful effect on presence are bordered in yellow, and included for broader interest. Outliers have been removed from each plot to improve clarity and interpretability of trends.

To understand which environmental features are associated with Snow Petrel presence, we implemented a binomial generalized additive model (GAM) using the ‘mgcvv’ package (Figure 3, Wood 2017). Generalized additive models are widely used in seabird macrohabitat studies (Certain et al. 2007; Gorta et al. 2019). Smoothing functions in GAMs enable the capture of ecological variability over very large spatial scales (Olivier and Wotherspoon 2005). This is valuable when considering marine habitat use of seabirds such as the Snow Petrel, which travels enormous distances throughout the year (Viola et al. 2023). Model parameters are further described in Table 1. Beyond initial correlation tests, all model outputs were assessed using the function *gam.check* within the ‘mgcv’ package. This function produces diagnostic information about model fit and convergence, and tests the null hypothesis that the basis dimension associated with a given variable is of sufficient size. For this approach, model parameters were deemed suitable when *p* > 0.05. The best model was chosen using Akaike's Information Criterion (AIC) (Akaike 2011).

#### 2.2.2 Question 2: How Do Varying Shipboard Survey Methods Affect Model Outputs?

Next, we extracted a subset of higher resolution seabird observation data (i.e., with complete bird count and observer effort) than the complete dataset. We used this data to understand the effect of accounting for effort and counts on region‐specific model outputs. More specifically, we used Snow Petrel data associated with the BROKE‐West voyage (Woehler et al. 2010). The total amount of along‐track survey effort associated with this data is 5653 km.

**FIGURE 4:**
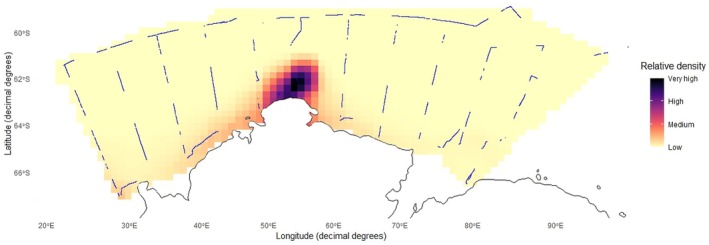
Snow Petrel ( *Pagodroma nivea* ) spatial density around Prydz Bay, Antarctica, during the BROKE‐West voyage. Areas subject to observer effort are depicted by blue lines. Density is indicated by cell colour where darker colours infer greater density.

To visualize spatial density patterns across the survey region, observation periods were treated as strip transects extending 300 m from the vessel, with no adjustments made for detectability related to distance, observer variation, or flock size. It was assumed that the probability of detecting Snow Petrels remained constant across the 300 m strip width. Continuous observation periods were divided into segments along the ship's track to provide independent density estimates of Snow Petrels, calculated as the number of individuals per segment divided by the segment area. Each segment was approximately 30 km in length. These segment‐based density estimates were then passed into a GAM to predict density estimates across the survey region and plotted with a soap‐film smoother (Figure 4).

Next, we generated two GAMs identical in nature except that one was effort‐quantified and used count data as the response variable (i.e., it used the raw BROKE‐West data); while the other response variable was down sampled to resemble the binomial structure (presence/absence) of the multi‐annual model used to answer *Question 1* (i.e., BROKE‐West data was used, but it was altered to mimic a lower resolution input).

Each model was built with the inclusion of all environmental variables (save for those that were colinear within the subset—i.e., bathymetry and sea‐surface height anomaly), but we used double‐penalty smoothers within ‘mgcv’ to perform model selection (i.e., to select which environmental variables were used). Explicitly, shrinkage smoothers in GAMs are special versions of standard basis types that undergo an Eigen decomposition when forming the penalty matrix. In this process, basis functions that are perfectly smooth receive zero eigenvalues (i.e., they do not change). The shrinkage smoother then adds a tiny value to these zero eigenvalues, causing them to be influenced by the usual penalty for wiggliness, which is used to determine smoothness. This method implies that wiggly functions should be reduced more than the smooth functions because the small addition to the zero‐eigenvalue terms makes them less affected by the wiggliness penalty than the other functions (Wood 2017).

However, when using a double‐penalty approach, the function introduces an additional penalty that specifically targets functions in the null space. This results in two penalties: (1) the standard wiggliness penalty, which affects functions in the range space, and (2) the shrinkage penalty, which impacts functions in the penalty null space. This second penalty allows the linear term to be shrunk, and together, both penalties can result in a smooth function being entirely removed from the model. The advantage of the double‐penalty approach is that it treats null space and range space functions equally in terms of shrinkage. Unlike the shrinkage smoother approach, which assumes that wiggly terms will be shrunk more than smooth terms, the double‐penalty approach does not make this assumption and allows all functions to be shrunk. The downside of the double‐penalty approach is that each smooth now requires the estimation of two ‘smoothness’ parameters: the usual smoothness parameter associated with the wiggliness penalty, and an additional smoothness parameter that controls the shrinkage for functions in the null space (Wood 2017). Roberts (2019), and Roberts et al. (2016) suggest that currently, there is little difference between the smoother methods, but the results of Marra and Wood (2011) suggest that the double penalty is slightly better—which was the driving point behind our decision to use a double‐penalty approach.

As the BROKE‐West data is count data with signs of overdispersion, we assigned a Tweedie family to the effort‐quantified model, and then a binomial family to the down sampled presence/absence model, as is recommended by the package developers (Wood 2017). The most suitable model fit (effort‐quantified, or presence/absence) was assessed using deviance explained values.

## 3 Results

### 3.1 Multi‐Annual Model Using the Complete Dataset (Question 1)

Between 1991 and 2006, Snow Petrel presence was correlated with bathymetry, sea‐ice coverage, wind, and sea‐surface height (*p* < 0.01, Table 2). Snow Petrel presence increased with decreasing sea‐surface height and higher wind speeds (up to a limit of 10 m/s, before presence decreased again). Presence slightly increased with shallower depths. Presence decreased slightly with decreasing sea‐ice coverage (Figure 3).

**TABLE 2:** Summary of the generalised additive model (GAM) describing environmental influence (significance) on Snow Petrel ( *Pagodroma nivea* ) presence. Significant variables are italicized.

Variable	Effective degrees of freedom	Reference degrees of freedom	*χ* ^2^	*p*
*Bathymetry*	*2.531*	*2.841*	*34.258*	*< 0.01*
*Sea ice coverage*	*2.976*	*2.999*	*425.629*	*< 0.01*
*Wind*	*2.681*	*2.917*	*29.890*	*< 0.01*
Eddy kinetic energy	1.002	1.004	0.282	0.598
*Sea surface height*	*1.800*	*2.143*	*38.226*	*< 0.01*
Sea surface height anomaly	2.399	2.718	8.606	0.026
Chlorophyll‐*a*	1.646	1.991	1.004	0.584
		**Deviance explained**		39.2%

Eddy kinetic energy, sea‐surface height anomaly, and chlorophyll‐*a* concentration were not significantly correlated with presence. However, trends suggest that presence decreases with increasing eddy kinetic energy; peaks with a lack of sea‐surface height anomaly; and increases with chlorophyll‐*a* concentration (Figure 3).

### 3.2 Weighted and Unweighted Models Using the Subset of Data From the BROKE‐West Voyage (Question 2)

Snow Petrel observations were most densely situated between longitudinal parallels 50° E and 60° E (Figure 4). Snow Petrels were most commonly observed near the coast, with densities decreasing towards northern latitudes and along the coast away from 50°–60° E (Figure 4).

**FIGURE 5:**
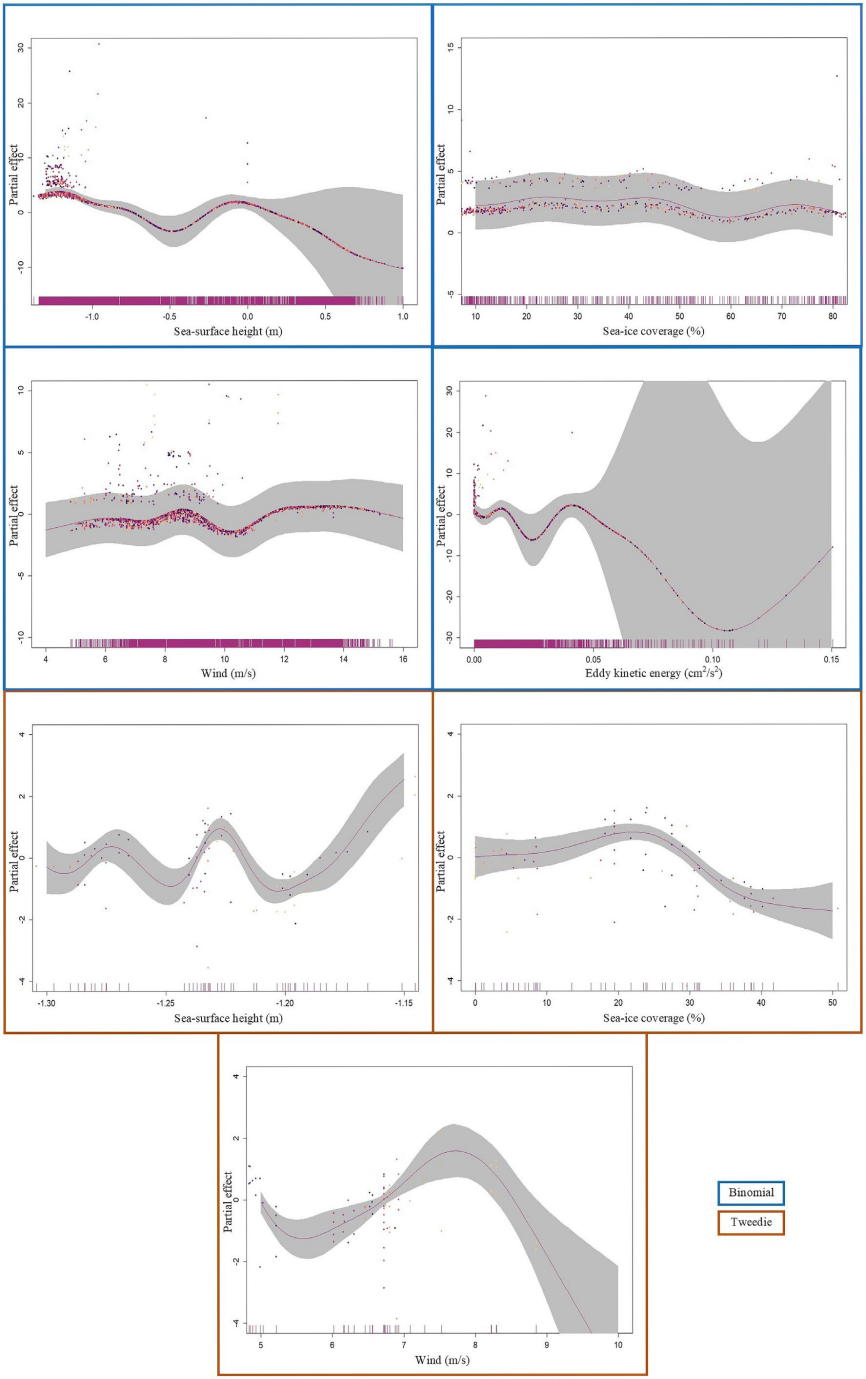
Relative likelihood of Snow Petrel ( *Pagodroma nivea* ) occurrence (partial effect) with regards to environmental variables as determined by a binomial (presence/absence data) generalised additive model (blue border), and a Tweedie (effort‐quantified count data) generalised additive model (orange border). Nonsignificant results have been omitted. Reprojections of the best fit output on the probability scale can be seen in appendices.

The effort‐quantified model (deviance explained = 80.8%) outperformed the presence/absence model (deviance explained = 42.3%). The effort‐quantified model identified sea‐ice concentration, wind speed, sea‐surface height, and chlorophyll‐*a* concentration as key environmental variables associated with Snow Petrel presence (Table 3, Figure 5, AppendixA7). The presence/absence model identified sea‐ice concentration, wind speed, sea‐surface height, and eddy kinetic energy as key environmental variables associated with Snow Petrel presence.

**TABLE 3:** Summary of the effort‐quantified (Tweedie) and presence/absence (binomial) generalised additive models (GAM) describing environmental correlation with Snow Petrel ( *Pagodroma nivea* ) presence. Significant variables are italicized.

Effort‐quantified				
Variable	Effective degrees of freedom	Reference degrees of freedom	*χ* ^2^	*p*
*Sea ice coverage*	*4.157*	*9*	*10.220*	*< 0.01*
*Wind*	*5.462*	*9*	*6.157*	*< 0.01*
Eddy kinetic energy	1.446e‐04	9	0.000	0.702
*Sea surface height*	*7.209*	*9*	*11.088*	*< 0.01*
Chlorophyll‐a	1.547e‐05	9	0.000	0.514
		**Deviance explained**		80.8%

Presence/absence				
Variable	Effective degrees of freedom	Reference degrees of freedom	*χ* ^2^	*p*
*Sea ice coverage*	*8.327*	*9*	*451.436*	*< 0.01*
*Wind*	*6.663*	*9*	*62.963*	*< 0.01*
*Eddy kinetic energy*	*5.933*	*9*	*37.585*	*< 0.01*
*Sea surface height*	*6.039*	*9*	*70.222*	*< 0.01*
Chlorophyll‐*a*	1.145	9	3.385	0.515
		**Deviance explained**		42.3%

## 4 Discussion

This study expands upon the limited understanding of Snow Petrel marine habitat use in East Antarctica (Viola et al. 2023) and contributes information that serves as an important baseline for the classification of seabirds according to the oceanographic properties of their at‐sea habitat (Ainley et al. 1984). Further, this work reconciles data from the past with contemporary knowledge, evaluates the performance of models with effort‐quantification, and demonstrates that survey‐based data is beneficial when modeling habitat for species in remote regions that are difficult to track or monitor.

### 4.1 Multi‐Annual, and Environmental Insights (Question 1)

Sea‐ice coverage was the environmental feature most strongly correlated with Snow Petrel presence (see highest *χ* ^2^ values in Table 2). This corroborates findings in other regions of Antarctica at varying spatial scales (Ainley and Jacobs 1981; Fraser and Ainley 1986; Woehler et al. 2010; Ainley et al. 2012, 2017). The Snow Petrel is an ice‐obligate species (Ainley et al. 2017) and anecdotally associates with open water near ice rather than the ice itself (Marchant and Higgins 1990). The ultimate driver behind Snow Petrel presence being associated with sea‐ice has been speculatively attributed to a physiological incapacity to travel beyond high latitudes (i.e., an inability to reach coastlines beyond Antarctica) (Griffiths 1983), though recent tracking studies have shown this is unlikely to be the case (Viola et al. 2023). In fact, the northernmost record of a Snow Petrel captured by our survey data occurs at 37°22′00.1″ S, 79°40′00.1″ E; a latitude that would easily constitute reaching coastlines beyond Antarctica if the longitude had been different.

While greater sea‐ice cover is associated with improved breeding success and fledgling body condition (Barbraud and Weimerskirch 2001; Olivier et al. 2005) it also negatively affects adult survival (Barbraud et al. 2000). In addition, the relationship between sea‐ice extent and Snow Petrel ecology is influenced by the seasonality of sea‐ice. Snow Petrel breeding effort and success at colonies near Casey Station are negatively affected by expansive sea‐ice during the preceding January–February (Austral summer), but positively affected by expansive sea‐ice during the preceding April–May (Austral winter) (Olivier et al. 2005). The slight increase in association found by our study between higher sea‐ice coverage and increased Snow Petrel presence (Figure 3) is unexplained, but is likely connected to a preference for foraging at the sea‐ice edge. Ultimately, the relationship between sea‐ice and Snow Petrel ecology warrants further investigation.

Bathymetry, wind, and sea‐surface height—all of which are associated with biological productivity (de Jong et al. 2013; Song et al. 2018; Bogumil et al. 2024)—were analogously correlated with the probability of Snow Petrel presence (see *χ* ^2^ values in Table 2). Each of these variables contributes to mixed layer dynamics within the ocean's water column (Smith 2022). Bathymetry is important because homogenous mixed layers can be considerably deeper than active mixing layers (Taylor and Ferrari 2011), which in turn cycle important nutrients through the water column in response to changing wind speeds or ice‐generated mixing and supply food for organisms at the bottom of the marine food web (Hunt et al. 2021; Smith 2022). Beyond driving physical ocean processes, wind can also introduce nutrients to the ocean via sediment resuspension (de Jong et al. 2013) and may introduce physical barriers associated with accessibility (i.e., birds may not be able to reach optimal foraging regions if flight is impaired by heavy winds). Additionally, the observed importance of low sea‐surface height can also be linked to ocean mixing and biological productivity as it is associated with oceanic surface pressure and other physical factors such as frontal systems and upwelling (Sokolov and Rintoul 2007).

Combined, these features facilitate macronutrient supply to regions around the Southern Ocean which drive biological productivity. Yet the two variables tested in our model which act as proxies for primary productivity—chlorophyll‐*a* concentration and eddy kinetic energy—were not associated with Snow Petrel presence (Table 2, Figure 3). Therefore, other mechanisms associated with bathymetry, wind, and sea‐surface height may also drive Snow Petrel at‐sea distribution. We believe that perhaps a combination of these variables in the right circumstances may specifically relate to the foraging niche of Snow Petrels. That is, areas subject to Snow Petrel foraging behavior may need a combination of high productivity (food availability) associated with chlorophyll‐*a* and eddies, but also highly dynamic ocean systems (transport) subject to wind/sea‐surface height (storms and upwellings). Disentangling this relationship is beyond the scope of our study, but with greater temporal and spatial data coverage in the future, it could be possible.

The Southern Ocean is renowned for being a high‐nutrient, low‐chlorophyll system (Uchida et al. 2019). So, it is not overly surprising that the observed effect of chlorophyll‐*a* concentration on the probability of Snow Petrel presence is nonsignificant (Table 2, Figure 3). However, if macronutrients (such as nitrate, phosphate, and silicate) were driving Snow Petrel presence, it is expected that a similar signal would be detected through high model importance of eddy kinetic energy: in general, eddies supply nutrients to the euphotic zone, which in turn nourish the phytoplankton containing chlorophyll‐*a* (Uchida et al. 2019). It is possible that the effects of chlorophyll‐*a* and eddy kinetic energy were downplayed by the model on account of the data collection technique. That is, Snow Petrel sightings were sparsely recorded alongside behavioral notes throughout the study period and were subsequently removed during the data cleaning process. If sightings were specifically associated with foraging activity, then the influence of both chlorophyll‐*a* concentration and eddy kinetic energy may have been more pronounced.

In addition to the survey method, the inherent difficulties of accurately capturing the nature of chlorophyll‐*a* concentrations in the Southern Ocean (Garcia et al. 2005) may have affected the model's capacity to estimate its importance. High densities of Snow Petrel prey items, such as Antarctic Krill ( *Euphausia superba* ) can rapidly deplete phytoplankton blooms, making the detection of chlorophyll‐*a* difficult (Cavan et al. 2019). However, the SeaWiFS data used is considered satisfactory for the study region and is relevant to the temporal span of the study period (Marrari et al. 2006).

It is also worth noting that while some studies incorporate a temporal lag to account for fluctuations in chlorophyll‐*a* concentration in response to solar irradiance, phytoplankton adapt to low solar irradiance environments by increasing the amount of chlorophyll per cell—obscuring interpretative power such that it is unclear whether photophysiological change or actual phytoplanktonic growth is occurring (Smith 2022). Coupled with mixed‐layer dynamics associated with sea ice, wind, and bathymetry (Smith 2022); as well as cloud cover obscuring satellite sensitivity for remotely sensed chlorophyll‐*a* (Moore and Abbott 2002), we believe that the monthly resolution chosen was best for this study. Explicitly, the model lost some granularity in determining the influence of chlorophyll‐*a*, but over the temporal span of the study period, we assumed that if important, the effect of chlorophyll‐*a* on the probability of Snow Petrel presence would still be detectable. It is possible that, despite chlorophyll‐*a* concentration being an important driver in many marine ecosystems (Suryan et al. 2012), much of the chlorophyll‐*a* present during winter is ice‐associated algae, and as such, is not detectable by satellite. Therefore, Snow Petrels may be associated with areas of the highest chlorophyll‐*a* concentration (with sea‐ice coverage, and distance to ice edge (Table 2, Figure 4) acting as a proxies), but the inability to observe these data via satellite affects model interpretation.

### 4.2 Comparing the Effect of Observer Effort on Model Outputs (Question 2)

Critically, at this point of the discussion, it is important to emphasize that the comparisons being made hereon are solely between the high‐resolution presence/absence (binomial) and effort‐quantified (Tweedie) models. These models use data from a much smaller spatial extent than the preceding multi‐annual, large‐scale binomial model we used to answer *Question 1* (explicitly, comparisons between which environmental features were correlated with Snow Petrel presence may be made between outputs from *Question 1* and *Question 2* with caution; but comparisons between trends for each feature cannot be made given the differences in volume, and spatiotemporal properties of each dataset).

The effort‐quantified model (deviance explained = 80.8%, tighter confidence intervals) outperformed the presence/absence model (deviance explained = 42.3%) indicating a better fit to the data. The environmental variables identified by the effort‐quantified model as associated with Snow Petrel presence were sea‐ice concentration, wind speed, and sea‐surface height. In contrast, the presence/absence model identified sea‐ice concentration, wind speed, sea‐surface height, and eddy kinetic energy as important predictors (Table 3, Figure 5, Appendix A7). The previous section of this discussion deals heavily with the interpretation of environmental variable correlation with Snow Petrel presence, but of interest with this subset are the variables identified by both models as significant—namely, sea‐ice concentration, wind speed, and sea‐surface height (Table 3, Figure 5).

Outputs from both models suggest that there is a slightly higher likelihood of Snow Petrel presence in weaker winds than in stronger winds. There are three ways to interpret this. The first is that Snow Petrels are less active in high winds—opting to bunker down when conditions become overly adverse. This is ecologically plausible, but probably not within the range of wind speeds listed. The study area is regularly subject to higher wind speeds, and Snow Petrels (like many other seabirds) become more active—wheeling up and down on the horizon (Ainley et al. 2015; Marchant and Higgins 1990; Menkhorst et al. 2017). What is more likely is that higher wind speeds are often associated with white‐caps and rougher sea conditions (Thorpe et al. 1987). This can make it more difficult to detect Snow Petrels (especially considering they are a small, all‐white bird that surface feeds near to the white‐caps of waves). It is therefore possible that this trend represents a detectability curve driven by observer ability, rather than an environmental correlation with Snow Petrel presence. The final interpretation is that the voyage during which these data were collected was not subjected to adverse conditions, and thus there is not enough of a spread in conditions for the model to make meaningful outputs.

With regards to sea‐surface height, Snow Petrel presence was strongly associated with negative values. As above, this could be associated with the small temporal span of data and subsequently related to the conditions encountered by the voyage. However, it is also possible Snow Petrels actively target areas of low sea‐surface height. These areas have been found to be important by tracking studies of Snow Petrels and were deemed representative of high biological activity (Viola et al. 2023).

Both models indicate a relatively neutral relationship between sea‐ice concentration and Snow Petrel presence. However, they differ in the range of sea‐ice concentrations considered. This variation stems from the effort‐corrected dataset, which excludes incidental sightings not recorded under the standardized survey protocol. Therewith, as eddy kinetic energy showed the weakest association with Snow Petrel presence in the presence/absence model output (see *χ* ^2^ values, Table 2), it is likely linked to areas with high sea‐ice concentration—a theory consistent with the general understanding that sea ice tends to suppress eddy activity in polar regions (Von Appen et al. 2022).

Reassuringly, both models identified similar trends with regard to the influence of wind and sea‐surface height on Snow Petrel presence. Had these been different, the only measure to compare would have been the deviance explained values—which would have introduced a level of doubt. Nevertheless, observed differences between the model outputs have potential implications for forecasting models—where inputs for training affect the model output (Arahal et al. 2002; Rasp and Thuerey 2021). Snow Petrel, and broader seabird community studies, could leverage our findings to understand how drastically changes in model structure—such as effort quantification—affect habitat suitability forecasts. However, for this sort of proposed study, sighting data with greater spatial independence is recommended, as predictive models struggle without considerable treatment when data is spatially autocorrelated (Stojanova et al. 2013). In this vein, we recommend greater spatiotemporal coverage of seabird surveys that account for observer effort going forward. One way to achieve this could be via enhanced collaboration between international Antarctic and Southern Ocean programs, but also by leveraging infrastructure for standardized opportunistic surveys (Viola et al. 2024).

### 4.3 A Broader Contextualization

As the Snow Petrel is a monotypic species (noting ongoing taxonomic discussion: Carrea et al. 2019; Kim and Kim 2020) of ecological importance (i.e., a Southern Ocean marine predator, Hindell et al. 2020) existing in an ecoregion with comparatively low vertebrate diversity (Olson et al. 2001; Eastman 2005), a sound understanding of its habitat is considered highly important. As mentioned previously, there are numerous legislative obligations within the Antarctic Treaty System that justify the continued monitoring and development of species‐specific information in Antarctica (Phillips et al. 2022).

When compared to other fulmarine petrels in the Southern Hemisphere, the dietary preferences of Snow Petrels are somewhat incongruous with heterospecifics: the two giant petrels are known scavengers (Marchant and Higgins 1990); Pintado Petrels and Antarctic Petrels tend to consume more krill than fish, whereas Snow Petrels and Southern Fulmars consume more fish than krill (Hodum and Hobson 2000); and Southern Fulmars consume less fish than Snow Petrels (Delord et al. 2016). Though such differences can be marginal (Hodum and Hobson 2000), it is likely—as seen in other, more temperate, seabird communities—that the dietary preferences of each species drive differences in habitat foci and therefore the environmental features each species associates with (Halpin et al. 2022).

Since the 20th century, shipboard surveys have played a key role in advancing our understanding of seabird ecology (Ainley et al. 2012). Nevertheless, regions such as East Antarctica remain relatively under‐surveyed, and the habitat preferences of many species breeding along the eastern coast are still poorly understood. This study provides valuable insights that facilitate comparisons between recent biologging data (Viola et al. 2023) and historical information that was previously inaccessible. Reassuringly, despite a temporal mismatch due to the seasonal nature of the data, the environmental variables most strongly associated with Snow Petrel presence in our model closely align with those identified in a biologging study that incorporated foraging behavior via state‐space modeling (Viola et al. 2023). Moreover, the patterns observed between Snow Petrel presence and individual environmental variables are broadly consistent with the findings of that study. Both studies identified positive associations with high sea‐ice concentration and negative associations with sea‐surface height. While bathymetry and wind were also linked to presence in both cases, the specific trends differed marginally between studies, likely due to the increased sea‐ice extent during the nonbreeding season (i.e., the period during which the biologging study occurred). We believe that the similarities between these studies suggest a consistent behavioral state, in which Snow Petrels target similar oceanic properties likely associated with foraging opportunities throughout the year. Future surveys could add greater certainty to such claims by incorporating behavioral information in their sighting data.

## 5 Conclusions

Despite relying on shipboard survey data, the variables associated with greater Snow Petrel presence within this study are similar to those identified in a much more recent biologging study (Viola et al. 2023). This supports the conclusion that Snow Petrels in East Antarctica are wide‐ranging generalists with variable movement strategies. Future work would benefit from a standardized approach to shipboard data collection, and the inclusion of behavioral annotations for all observations.

## Author Contributions

**Benjamin Viola:** conceptualization (equal), data curation (equal), formal analysis (lead), investigation (lead), methodology (lead), project administration (lead), software (lead), writing – original draft (lead), writing – review and editing (lead). **Luke Halpin:** formal analysis (equal), investigation (equal), methodology (equal), validation (equal), writing – review and editing (equal). **Denisse Fierro‐Arcos:** formal analysis (equal), investigation (equal), methodology (equal), validation (equal), writing – review and editing (equal). **Toby Travers:** formal analysis (equal), investigation (equal), methodology (equal), validation (equal), writing – review and editing (equal). **Louise Emmerson:** conceptualization (equal), investigation (equal), project administration (equal), supervision (equal), writing – review and editing (equal). **Colin Southwell:** conceptualization (equal), investigation (equal), methodology (equal), project administration (equal), supervision (equal), writing – review and editing (equal). **Patti Virtue:** formal analysis (equal), investigation (equal), project administration (equal), supervision (equal), writing – review and editing (equal). **Natalie Kelly:** conceptualization (equal), data curation (equal), formal analysis (equal), investigation (equal), methodology (equal), project administration (equal), supervision (equal), writing – review and editing (equal). **Stuart Corney:** conceptualization (equal), formal analysis (equal), investigation (equal), methodology (equal), project administration (equal), supervision (lead), writing – review and editing (equal).

## Conflicts of Interest

The authors declare no conflicts of interest.

## Supporting information


**Data S1:** ece371871‐sup‐0001‐DataS1.csv.

## Data Availability

We have uploaded the data used for this study as Supporting Information (.csv) to this manuscript. Also, all shipboard survey data (from various voyages) are openly available from the Australian Antarctic Data Centre (https://data.aad.gov.au/).
